# An improved approximation algorithm for the reversal and transposition distance considering gene order and intergenic sizes

**DOI:** 10.1186/s13015-021-00203-7

**Published:** 2021-12-29

**Authors:** Klairton L. Brito, Andre R. Oliveira, Alexsandro O. Alexandrino, Ulisses Dias, Zanoni Dias

**Affiliations:** 1 Institute of Computing, University of Campinas, 1251 Albert Einstein Ave., 13083-852 Campinas, Brazil; 2 School of Technology, University of Campinas, 1888 Paschoal Marmo St., 13484-332 Limeira, Brazil

**Keywords:** Genome rearrangement events, Intergenic regions, Approximation algorithms

## Abstract

**Background:**

In the comparative genomics field, one of the goals is to estimate a sequence of genetic changes capable of transforming a genome into another. Genome rearrangement events are mutations that can alter the genetic content or the arrangement of elements from the genome. Reversal and transposition are two of the most studied genome rearrangement events. A reversal inverts a segment of a genome while a transposition swaps two consecutive segments. Initial studies in the area considered only the order of the genes. Recent works have incorporated other genetic information in the model. In particular, the information regarding the size of intergenic regions, which are structures between each pair of genes and in the extremities of a linear genome.

**Results and conclusions:**

In this work, we investigate the sorting by intergenic reversals and transpositions problem on genomes sharing the same set of genes, considering the cases where the orientation of genes is known and unknown. Besides, we explored a variant of the problem, which generalizes the transposition event. As a result, we present an approximation algorithm that guarantees an approximation factor of 4 for both cases considering the reversal and transposition (classic definition) events, an improvement from the 4.5-approximation previously known for the scenario where the orientation of the genes is unknown. We also present a 3-approximation algorithm by incorporating the generalized transposition event, and we propose a greedy strategy to improve the performance of the algorithms. We performed practical tests adopting simulated data which indicated that the algorithms, in both cases, tend to perform better when compared with the best-known algorithms for the problem. Lastly, we conducted experiments using real genomes to demonstrate the applicability of the algorithms.

## Background

In the comparative genomics field, there are many ways to compare genomic features like DNA sequence, gene order, and genomic landmarks from different organisms. Genome rearrangement events are mutations that affect large stretches of the DNA sequence. Determining the shortest sequence of such events that can transform one genome into another is widely used as a metric to study evolutionary relationships among organisms, and to explain biological similarities and differences as well. The reversal and transposition are two of the most studied genome rearrangement events in the literature [1–3]. A reversal inverts a segment of a genome, and a transposition moves a segment of a genome to another position.

One way to represent a genome is by using the gene order as the only genomic trait, which can be encoded as a sequence of elements, where each element represents a gene. When the compared genomes share the same set of genes and do not have replicated genes, we model them as permutations of natural numbers, such that each number in the sequence appears once. Furthermore, if the orientation of the genes is known, a plus or a minus sign (+ or −) is assigned to each element of the permutation to indicate its orientation, and we say that the permutation is a signed permutation. Otherwise, signs are omitted and the permutation is called unsigned.

It is always possible to map the target genome in a permutation such that the elements are in increasing order. This permutation is called by identity permutation and denoted as $\iota =(1~2~\dots ~n)$ and $\iota =({+1}~{+2}~\dots ~{+n})$, considering unsigned and signed cases, respectively. Therefore, the transformation from a source genome to a target genome can be seen as a sorting problem.

First studies in the genome rearrangement field considered a single type of rearrangement events, which led to solutions specific to that type. In particular, the reversal event leads to the sorting by reversals problem, which has a polynomial-time algorithm on signed permutations [1], whereas it is NP-hard on unsigned permutations [4] and the best algorithm has an approximation factor of 1.375 [2]. The transposition event leads to the sorting by transpositions problem, which is NP-hard [5] and the best algorithm has an approximation factor of 1.375 [3]. By allowing both reversal and transposition we have the sorting by reversals and transpositions problem, which is NP-hard on signed and unsigned permutations [6]. The best algorithms have approximation factors of 2 [7] and $2.8334+\epsilon$ [8, 9] for signed and unsigned permutations, respectively.

The gene order was fundamental to the initial development of rearrangement distance models. However, recent studies indicate that incorporating another genetic information apart from the gene order could generate more realistic models [10, 11]. In particular, the information regarding the size of intergenic regions (structures with a specific number of nucleotides between each pair of genes and in the extremities of genomes) was incorporated into the mathematical models.

The Double Cut and Join (DCJ) is a rearrangement event that cuts the genome in two points and reassembles the stretches following a predetermined criterion. The problem of sorting by dcjs with intergenic regions is NP-hard [12], but one can find a polynomial-time algorithm when DCJs are used together with insertions and deletions on intergenic regions [13]. The Block-Interchange is a rearrangement event that swaps the position of two segments (not necessarily consecutive) of the genome. The sorting by intergenic block-interchange problem has a 2-approximation algorithm [14] and its complexity is unknown. Considering the reversal event, we have the sorting by intergenic reversals problem, which is NP-hard on signed and unsigned permutations [15, 16] and the best algorithms have approximation factors of 2 [15] and 4 [16], respectively. The sorting by intergenic transpositions is NP-hard and the best algorithm has an approximation factor of 3.5 [17]. The sorting by intergenic reversals and transpositions (SbIRT) is NP-hard on signed and unsigned permutations [16, 17] and the best algorithms have the approximation factors of 3 [17] and 4.5 [16], respectively. The ${\textsc {SbIRT}}$ problem with the generalized definition of the transposition event on signed permutations has an approximation algorithm with a factor of 2.5 [17].

The ${\textsc {SbIRT}}$ problem with an additional constraint that limits the number of genes affected by each operation, called super short operations, was investigated [18]. On signed permutations it was proposed a 5-approximation algorithm, while for unsigned permutations it was proposed a 3-approximation algorithm.

In this work, we investigate the ${\textsc {SbIRT}}$ problem on signed and unsigned permutations. For the unsigned case, we present an improved algorithm based on intergenic breakpoints that guarantees an approximation factor of 4. We also show a 3-approximation algorithm for the ${\textsc {SbIRT}}$ problem on unsigned permutations considering the generalized definition of the transposition event. For the signed case, we show approximation algorithms with the same approximation factors as in the unsigned cases. Although the theoretical approximations for the signed case are superior to the previously known results, the tests with simulated data pointed that our algorithms tend to provide better practical results. We propose a greedy strategy to improve the algorithms’ performance and tested them using simulated and real data.

This manuscript is organized as follows. "Definitions" section presents concepts and definitions used throughout the paper. "Theoretical results" section shows a lower bound and the approximation algorithms for the ${\textsc {SbIRT}}$ problem. "Practical results" section shows the experiments using real and simulated data. "Conclusion" section concludes the paper and introduces future directions.

## Definitions

The problem we investigate uses information about source and target genomes. We assume that both genomes share the same set of genes and there are no replicated genes. Thus, given a linear genome ${\mathcal {G}}=(i_1,g_1,i_2,g_2,\dots ,i_n,g_n,i_{n+1})$ with *n* genes and $n+1$ intergenic regions, we use (i) a permutation $\pi$, representing the order of the genes, and (ii) a list of non-negative integer numbers $\breve{\pi }$, representing the sizes of intergenic regions. If the orientation of the genes is known, a “$+$” or “−” sign is associated with each element of the permutation $\pi$ to indicate its orientation. We use $\pi _i$, $1 \le i \le n$, to denote the element in position *i* of $\pi$. Similarly, we denote by $\breve{\pi }_i$ the size of the intergenic region in the left of $\pi _i$. The intergenic region $\breve{\pi }_{n+1}$ is on the right of $\pi _n$.

**Fig. 1:**
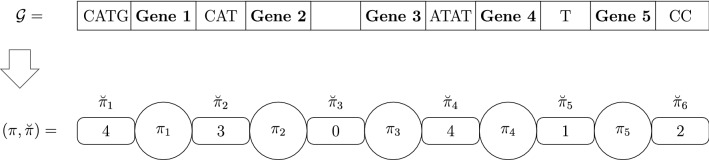
On the top we have a fictitious genome ${\mathcal {G}}$, with 5 genes and an intergenic region between each pair of genes and also in the extremities of ${\mathcal {G}}$. Each intergenic region has a specific number of nucleotides (represented by the letters A, C, G, or T). At the bottom, we have the genome representation for the ${\textsc {SbIRT}}$ problem, using a permutation $\pi$ and a list $\breve{\pi }$, representing the order of the genes and the sizes of the intergenic regions, respectively. Observe that for each intergenic region $\breve{\pi }_i$ we have the information about the number of nucleotides inside it

For convenience, we map the genes from the target genome to the identity permutation $\iota =(1~2~\dots ~n)$ for the case where the orientation of the genes is unknown and $\iota =({+1}~{+2}~\dots ~{+n})$ otherwise. The permutation $\pi$ of the source genome can be mapped according to how we assigned elements to genes while mapping the target genome to the identity permutation, so the source and target genomes are represented as $(\pi ,\breve{\pi })$ and $(\iota ,\breve{\iota })$, respectively. Since the identity permutation is fixed given the size of the genomes, an instance for the ${\textsc {SbIRT}}$ problem is composed by $(\pi ,\breve{\pi },\breve{\iota })$. Figure 1 shows the representation of a genome ${\mathcal {G}}$ as $(\pi ,\breve{\pi })$.

From now on, we will refer to an instance $(\pi ,\breve{\pi },\breve{\iota })$ of the ${\textsc {SbIRT}}$ problem in which the orientation of the genes is known and unknown by signed and unsigned instances, respectively. In this work, initially, we consider two rearrangement events: intergenic reversal and intergenic transposition. In the following, we formally describe them and show how they affect a given genome representation.

### Definition 2.1

Given a genome $(\pi ,\breve{\pi })$, let *i*, *j*, *x*,  and *y* be integers such that $1 \le i \le j \le n$, $0 \le x \le \breve{\pi }_i$, and $0 \le y \le \breve{\pi }_{j+1}$. An intergenic reversal $\rho ^{(i, j)}_{(x, y)}$ applied to $(\pi ,\breve{\pi })$ splits the intergenic regions $\breve{\pi }_i$ (into $x|x^{\prime }$) and $\breve{\pi }_{j+1}$ (into $y|y^{\prime }$), where $x^{\prime }=\breve{\pi }_i-x$ and $y^{\prime }=\breve{\pi }_{j+1}-y$, and it reverses the sequence $(x^\prime ,\pi _i,\breve{\pi }_{i+1},\dots ,\breve{\pi }_j,\pi _j,y)$, generating $(\pi ^{\prime },\breve{\pi }^{\prime })$ as follows:

$$\begin{aligned} (\pi ,\breve{\pi })= & {} (\dots \underline{\breve{\pi }_i,\pi _i,\breve{\pi }_{i+1}, \dots ,\breve{\pi }_{j},\pi _{j},\breve{\pi }_{j+1}},\dots )\\ (\pi ^{\prime },\breve{\pi }^{\prime })= & {} (\dots ,\underline{\breve{\pi }^{\prime }_i,\pi _j,\breve{\pi }_{j},\dots ,\breve{\pi }_{i+1},\pi _{i}, \breve{\pi }^{\prime }_{j+1}},\dots ), \end{aligned}$$

such that $\breve{\pi }^{\prime }_i= x + y$ and $\breve{\pi }^{\prime }_{j+1} = x^{\prime } + y^{\prime }$. If the orientation of the genes is known, the signs of the genes in the affected segment are flipped as follows:

$$\begin{aligned} (\pi ,\breve{\pi })= & {} (\dots \underline{\breve{\pi }_i,+\pi _i,\breve{\pi }_{i+1}, \dots ,\breve{\pi }_{j},+\pi _{j},\breve{\pi }_{j+1}},\dots )\\ (\pi ^{\prime },\breve{\pi }^{\prime })= & {} (\dots ,\underline{\breve{\pi }^{\prime }_i, -\pi _j,\breve{\pi }_{j},\dots ,\breve{\pi }_{i+1},-\pi _{i},\breve{\pi }^{\prime }_{j+1}},\dots ), \end{aligned}$$

### Definition 2.2

Given a genome $(\pi ,\breve{\pi })$, let *i*, *j*, *k*, *x*, *y*,  and *z* be integers such that $1 \le i< j < k \le n+1$, $0 \le x \le \breve{\pi }_i$, $0 \le y \le \breve{\pi }_j$ and $0 \le z \le \breve{\pi }_k$. An intergenic transposition $\tau ^{(i,j,k)}_{(x,y,z)}$ applied to $(\pi ,\breve{\pi })$ splits the intergenic regions $\breve{\pi }_i$ (into $x|x^{\prime }$), $\breve{\pi }_{j}$ (into $y|y^{\prime }$), and $\breve{\pi }_k$ (into $z|z^{\prime }$), where $x^{\prime }=\breve{\pi }_i-x$, $y^{\prime }=\breve{\pi }_j-y$, and $z^{\prime }=\breve{\pi }_k-z$, and swaps the adjacent segments $(x^{\prime },\pi _i,\breve{\pi }_{i+1},\dots ,\breve{\pi }_{j-1},\pi _{j-1},y)$ and $(y^{\prime },\pi _j,\breve{\pi }_{j+1},\dots ,\breve{\pi }_{k-1},\pi _{k-1},z)$, generating $(\pi ^{\prime },\breve{\pi }^{\prime })$ as follows:

$$\begin{aligned} (\pi ,\breve{\pi })= & {} (\dots ,\underline{\breve{\pi }_{i},\pi _i,\dots ,\pi _{j-1}, \breve{\pi }_{j},\pi _j,\dots ,\pi _{k-1},\breve{\pi }_{k}},\dots )\\ (\pi ^{\prime },\breve{\pi }^{\prime })= & {} (\dots ,\underline{\breve{\pi }^{\prime }_{i} ,\pi _j,\dots ,\pi _{k-1},\breve{\pi }^{\prime }_{i+k-j},\pi _i,\dots ,\pi _{j-1}, \breve{\pi }^{\prime }_{k}},\dots ), \end{aligned}$$

such that $\breve{\pi }^{\prime }_{i} = x + y^{\prime }$, $\breve{\pi }^{\prime }_{i+k-j} = z + x^{\prime }$, and $\breve{\pi }^{\prime }_{k} = y + z^{\prime }$.

Given a genome $(\pi ,\breve{\pi })$ and an operation $\gamma$, $(\pi ,\breve{\pi }) \cdot \gamma$ represents the operation $\gamma$ applied on $(\pi ,\breve{\pi })$. Similarly, given a sequence of operations $S_\gamma = (\gamma _1,\ldots ,\gamma _k)$, we use $(\pi ,\breve{\pi }) \cdot S_\gamma$ to denote $(\pi ,\breve{\pi }) \cdot \gamma _1 \ldots \gamma _k$. We hereafter refer to intergenic reversals and intergenic transpositions simply as reversals and transpositions, respectively. Note that both reversal and transposition are conservative events, i.e., they do not insert or remove genes nor nucleotides. Thus, an instance $(\pi ,\breve{\pi },\breve{\iota })$ from the ${\textsc {SbIRT}}$ problem is valid if the following equality is satisfied:

$$\begin{aligned} \sum _{\breve{\pi }_i \in \breve{\pi }} \breve{\pi }_i = \sum _{\breve{\iota }_i \in \breve{\iota }} \breve{\iota }_i. \end{aligned}$$

Given an instance $I=(\pi ,\breve{\pi },\breve{\iota })$ of the ${\textsc {SbIRT}}$ problem, the minimum number of operations needed to transform $(\pi ,\breve{\pi })$ into $(\iota ,\breve{\iota })$ is called the distance and is denoted by $d_{{\textsc {SbI}}\overline{\text {R}}{\textsc {T}}}(I)$ and $d_{{\textsc {SbIRT}}}(I)$ for the signed and unsigned cases, respectively. The extended form of $\pi$ is obtained by adding the elements $\pi _0 = 0$ and $\pi _{n+1} = (n+1)$ at the beginning and at the end of $\pi$, respectively. We hereafter assume that permutations are in extended form, and we refer to them simply as permutations. Following, we present concepts and definitions that are used in previous works [16] regarding the ${\textsc {SbIRT}}$ problem.

### Definition 2.3

Given an unsigned instance $I=(\pi ,\breve{\pi },\breve{\iota })$ of the ${\textsc {SbIRT}}$ problem, a pair of elements $(\pi _i,\pi _{i+1})$, such that $0 \le i \le n$, is an intergenic breakpoint type one if one of the following cases occur:

- $$|\pi _{i+1} - \pi _i| \ne 1$$.
- $|\pi _{i+1} - \pi _i| = 1$ and $\breve{\pi }_{i+1} \ne \breve{\iota }_{x}$, such that $x = \max (\pi _i,\pi _{i+1})$.

### Definition 2.4

Given an unsigned instance $I=(\pi ,\breve{\pi },\breve{\iota })$ of the ${\textsc {SbIRT}}$ problem, a pair of elements $(\pi _a,\pi _{b})$ is an intergenic adjacency if $|a-b|=1$ and $(\pi _{\min (a,b)},\pi _{\max (a,b)})$ is not an intergenic breakpoint type one.

In other words, an intergenic breakpoint type one indicates a region that must be affected by a rearrangement event to fix the order of the genes or the size of the intergenic region to reach the target genome. On the other hand, an intergenic adjacency indicates a pair of genes that are consecutive in the target genome and the intergenic regions between them have the same size. From now on, we will refer to intergenic breakpoint and intergenic adjacency as breakpoint and adjacency, respectively.

### Definition 2.5

A breakpoint type one $(\pi _i,\pi _{i+1})$, such that $|\pi _{i+1} - \pi _i| = 1$, is overcharged if $\breve{\pi }_{i+1} > \breve{\iota }_{x}$, such that $x = \max (\pi _i,\pi _{i+1})$, and undercharged otherwise.

### Definition 2.6

A pair of breakpoints type one $(\pi _{i},\pi _{i+1})$ and $(\pi _{j},\pi _{j+1})$ is connected if the following conditions are met:

1. The pair of elements $(\pi _{i},\pi _{i+1})$, $(\pi _{j},\pi _{j+1})$, $(\pi _{i},\pi _{j})$, $(\pi _{i},\pi _{j+1})$, $(\pi _{i+1},\pi _{j})$, or $(\pi _{i+1},\pi _{j+1})$ are consecutive in $\iota$ and do not form an adjacency in $(\pi ,\breve{\pi })$.
2. $\breve{\pi }_{i+1} + \breve{\pi }_{j+1} \ge \breve{\iota }_{k}$, such that $\breve{\iota }_{k}$ is the intergenic region size between the consecutive elements (from condition 1) in $\iota$.

A pair of connected breakpoints indicate that it is possible to form an adjacency using only the nucleotides from the intergenic regions of the two breakpoints. Note that a pair of connected breakpoints has at least one pair of consecutive elements between $\pi _{i}$, $\pi _{i+1}$, $\pi _{j}$, and $\pi _{j+1}$. Besides, the number of nucleotides in both breakpoints ($\breve{\pi }_{i+1} + \breve{\pi }_{j+1}$) is at least the size of the intergenic region between the consecutive elements in $\iota$.

Now, we introduce new definitions which are used to derive the results.

### Definition 2.7

A breakpoint type one $(\pi _i, \pi _{i+1})$ is called hard if it is overcharged or undercharged and soft otherwise.

Note that in hard breakpoints the pair of genes are consecutive in the target genome, but the intergenic region between them is not the same as in the target genome.

### Definition 2.8

A pair of breakpoints type one $(\pi _{i},\pi _{i+1})$ and $(\pi _{j},\pi _{j+1})$ is called softly connected if they are connected and both breakpoints are soft.

### Definition 2.9

A hard breakpoint $(\pi _i, \pi _{i+1})$ is called super hard if one of the following cases occur:

- $$i = 0$$ or $$i = n$$.
- $(\pi _{i-1}, \pi _{i})$ or $(\pi _{i+1}, \pi _{i+2})$ is a hard breakpoint or an adjacency.

Note that a super hard breakpoint is in one of the extremities of the genome, or immediately before or after the breakpoint exists a hard breakpoint or an adjacency.

### Definition 2.10

Given an unsigned instance $I=(\pi ,\breve{\pi },\breve{\iota })$, strips are maximal sequences of consecutive elements of $\pi$ without soft breakpoints. A strip with only one element $\pi _i$ is called a singleton, and it is defined as increasing if $i \in \{0, (n+1)\}$, and as decreasing otherwise. A strip with more than one element is called increasing if its elements form an increase sequence; and it is called decreasing otherwise.

For a signed instance of the ${\textsc {SbIRT}}$ problem, we have the following definition:

### Definition 2.11

Given a signed instance $I=(\pi ,\breve{\pi },\breve{\iota })$ of the ${\textsc {SbIRT}}$ problem, a pair of elements $(\pi _i,\pi _{i+1})$, such that $0 \le i \le n$, is an intergenic breakpoint type two if one of the following cases occur:

- $$\pi _{i+1} - \pi _i \ne 1$$.
- $\pi _{i+1} - \pi _i = 1$ and $\breve{\pi }_{i+1} \ne \breve{\iota }_{x}$, such that $x = \max (|\pi _i|,|\pi _{i+1}|)$.

Given an unsigned instance $(\pi , \breve{\pi },\breve{\iota })$ of the ${\textsc {SbIRT}}$ problem, the total number of hard and soft breakpoints are denoted by $b_h(\pi , \breve{\pi },\breve{\iota })$ and $b_s(\pi , \breve{\pi },\breve{\iota })$, respectively, and the total number of breakpoints type one is denoted by $b_{1}(\pi , \breve{\pi },\breve{\iota }) = b_h(\pi , \breve{\pi },\breve{\iota }) + b_s(\pi , \breve{\pi },\breve{\iota })$. The variation in the number of breakpoints type one after applying a rearrangement event $\gamma$ to $(\pi ,\breve{\pi })$ is denoted by $\Delta b_1(\pi , \breve{\pi },\breve{\iota },\gamma ) = b_1(\pi ^{\prime }, \breve{\pi }^{\prime },\breve{\iota }) - b_1(\pi , \breve{\pi },\breve{\iota })$, where $(\pi ^{\prime }, \breve{\pi }^{\prime }) = (\pi , \breve{\pi }) \cdot \gamma$. Similarly, given a signed instance $(\pi , \breve{\pi },\breve{\iota })$ of the ${\textsc {SbIRT}}$ problem, the total number of breakpoints type two is denoted by $b_{2}(\pi , \breve{\pi },\breve{\iota })$ and the variation in the number of breakpoints type two after applying a rearrangement event $\gamma$ to $(\pi ,\breve{\pi })$ is denoted by $\Delta b_2(\pi , \breve{\pi },\breve{\iota },\gamma ) = b_2(\pi ^{\prime }, \breve{\pi }^{\prime },\breve{\iota }) - b_2(\pi , \breve{\pi },\breve{\iota })$, where $(\pi ^{\prime }, \breve{\pi }^{\prime }) = (\pi , \breve{\pi }) \cdot \gamma$.

### Remark 2.1

The only unsigned instance *I* of the ${\textsc {SbIRT}}$ problem such that $b_1(I) = 0$ is $(\iota ,\breve{\iota },\breve{\iota })$. Similarly, the only signed instance $I^{\prime }$ of the ${\textsc {SbIRT}}$ problem, such that $b_2(I^{\prime }) = 0$ is $(\iota ,\breve{\iota },\breve{\iota })$. Thus, to transform $(\pi , \breve{\pi })$ into $(\iota ,\breve{\iota })$ it is necessary to remove all the breakpoints of an instance.

**Fig. 2:**
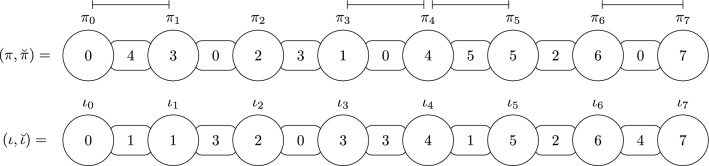
An instance $I=(\pi ,\breve{\pi },\breve{\iota })$, where $\pi =(0,3,2,1,4,5,6,7), \breve{\pi }=(4,0,3,0,5,2,0),$ and $\breve{\iota }=(1,3,0,3,1,2,4)$. On top we have the source genome $(\pi ,\breve{\pi })$ and in the bottom the target genome $(\iota ,\breve{\iota })$. Breakpoints are indicated above the source genome. Note that $b_h(I)=2$ and $b_s(I)=2$, so $b(I) = 4$. The hard breakpoints $(\pi _4,\pi _5)$ and $(\pi _6,\pi _7)$ are overcharged and undercharged, respectively. Breakpoints $(\pi _0,\pi _1)$ and $(\pi _3,\pi _4)$ are soft. The breakpoints $(\pi _0,\pi _1)$ and $(\pi _6,\pi _7)$ are connected, while $(\pi _3,\pi _4)$ and $(\pi _6,\pi _7)$ are not. Besides, the pair of breakpoints $(\pi _0,\pi _1)$ and $(\pi _3,\pi _4)$ are softly connected. The instance *I* has the increasing strips $(\pi _0)$ and $(\pi _4,\pi _5,\pi _6,\pi _7$), and the decreasing strip $(\pi _1,\pi _2,\pi _3)$

Figure 2 shows the concepts using a representation of the source and target genomes.

## Theoretical results

In this section, we show lower bounds and present approximation algorithms for both cases of the ${\textsc {SbIRT}}$ problem. We start by showing how many breakpoints a reversal and a transposition can remove in the best scenario.

### Lemma 3.1

*Given an unsigned instance* $I_1=(\pi , \breve{\pi },\breve{\iota })$ *and a signed instance* $I_2=(\pi , \breve{\pi },\breve{\iota })$ *of the* ${\textsc {SbIRT}}$ *problem,* $\Delta b_1(I_1,\rho ) \ge -2$ *and* $\Delta b_2(I_2,\rho ) \ge -2$ *for any reversal* $\rho ^{(i,j)}_{(x,y)}$*, respectively.*

### Proof

Recall that a reversal affects two pair of consecutive elements of $\pi$. In the best case, $(\pi _{i-1},\pi _{i})$ and $(\pi _{j},\pi _{j+1})$ are breakpoints, and the reversal $\rho ^{(i,j)}_{(x,y)}$ removes them. $\square$

### Lemma 3.2

*Given an unsigned instance* $I_1=(\pi , \breve{\pi },\breve{\iota })$ *and a signed instance* $I_2=(\pi , \breve{\pi },\breve{\iota })$ *of the* ${\textsc {SbIRT}}$ *problem,* $\Delta b_1(I_1,\tau ) \ge -3$ *and* $\Delta b_2(I_2,\tau ) \ge -3$ *for any transposition* $\tau ^{(i,j,k)}_{(x,y,z)}$*, respectively.*

### Proof

The proof is similar to the one described in Lemma 3.1, and considering that a transposition can affect up to three breakpoints. $\square$

Using the above lemmas we define a lower bound for the ${\textsc {SbIRT}}$ problem.

### Proposition 3.1

(Proposition 7 [16]) *Given an unsigned instance* $I=(\pi , \breve{\pi },\breve{\iota })$*,* $d_{{\textsc {SbIRT}}}(I) \ge \frac{b_1(I)}{3}$.

### Proposition 3.2

*Given a signed instance* $I=(\pi , \breve{\pi },\breve{\iota })$*,* $d_{{\textsc {SbI}}\overline{\text {R}}{\textsc {T}}}(I) \ge \frac{b_2(I)}{3}$.

### Proof

Directly by Remark 2.1 and lemmas 3.1 and 3.2. $\square$

### Approximation algorithms for the unsigned case of the ${\textsc {SbIRT}}$ problem

In this section, we investigate the unsigned case of the ${\textsc {SbIRT}}$ problem and present a 4-approximation algorithm considering the reversal and transpositions events. Besides, we show a 3-approximation algorithm incorporating a generalized definition of the transposition event. We show a sequence of lemmas that will be used by the algorithms as subroutines.

#### Lemma 3.3

(Lemma 19 [16]) *It is possible to perform any redistribution of nucleotides within intergenic regions* $\breve{\pi }_i$*,* $\breve{\pi }_j$*, and* $\breve{\pi }_k$ *using two consecutive transpositions in the format:*

$$\begin{aligned} (\pi ,\breve{\pi })\cdot \tau ^{(i,j,k)}_{(\varphi _i,\varphi _j,\varphi _k)} \cdot \tau ^{(i,i+k-j,k)}_{(\varphi ^\prime _i,\varphi ^\prime _{i+k-j},\varphi ^\prime _k)}. \end{aligned}$$

In this section, we will refer to breakpoint type one simply as a breakpoint. Now let us show how to remove breakpoints from an unsigned instance depending on how many overcharged breakpoints an instance has.

#### Lemma 3.4

*Given an unsigned instance* $(\pi ,\breve{\pi },\breve{\iota })$ *for the* ${\textsc {SbIRT}}$ *problem, if there are at least two overcharged breakpoints then there exists a sequence of two transpositions that removes at least two breakpoints.*

#### Proof

First note that a third breakpoint must exist in $(\pi ,\breve{\pi },\breve{\iota })$, otherwise the total number of nucleotides within intergenic regions of the source genome would be greater than the number of nucleotides within intergenic regions of the target genome. By Lemma 3.3, it is possible to make a redistribution of nucleotides within three intergenic regions using two consecutive transpositions. Without loss of generality, assume that two of these intergenic regions are between the two overcharged breakpoints, and that the third intergenic region is between an existing third breakpoint. In this case, the extra nucleotides from the two overcharged breakpoints are moved to the third breakpoint, and the lemma follows. $\square$

#### Lemma 3.5

*Given an unsigned instance* $(\pi ,\breve{\pi },\breve{\iota })$ *for the* ${\textsc {SbIRT}}$ *problem, if there is a pair of softly connected breakpoints then there exists a reversal or a transposition that removes at least one breakpoint.*

#### Proof

Brito et. al. [16, lemmas 14 and 20] showed how to remove a breakpoint from a pair of connected breakpoints. In particular, when both breakpoints $(\pi _i, \pi _{i+1})$ and $(\pi _j, \pi _{j+1})$ are soft, we have one of the following three possibilities to form at least one adjacency from them:

- Case 1: $(\pi _i, \pi _j)$ or $(\pi _{i+1}, \pi _{j+1})$ are consecutive in $\iota$.
- Case 2: $(\pi _{i+1}, \pi _{j})$ are consecutive in $\iota$.
- Case 3: $(\pi _{i}, \pi _{j+1})$ are consecutive in $\iota$.

For each one of the cases, a reversal or a transposition can be applied to remove at least one breakpoint, and the lemma follows. $\square$

**Fig. 3:**
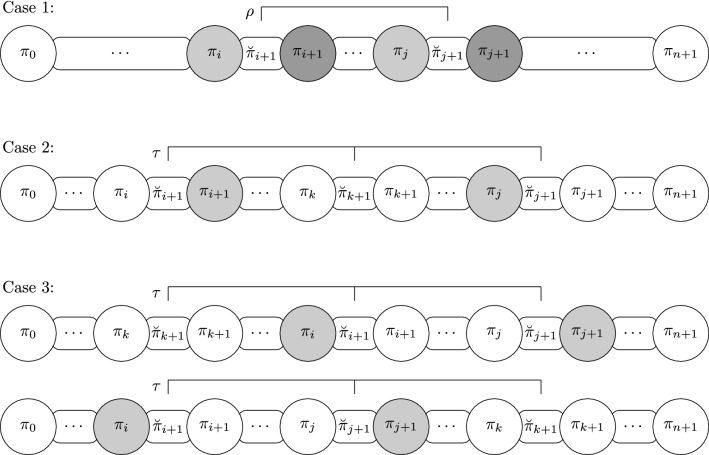
The possibilities that can arise when a pair of softly connected breakpoints exists. In this case, one operation can be applied to remove at least one breakpoint. The pair of elements that are consecutive in the identity permutation is represented with a grayscale color

Figure 3 shows, for each case in Lemma 3.5, a reversal or a transposition that can be applied to remove at least one breakpoint. In Case 3, a transposition is applied to the pair of soft breakpoints and in a third breakpoint, which can be located before or after the pair of soft breakpoints.

#### Remark 3.1

Note that Case 2 of Lemma 3.5 is the only one in which a hard breakpoint can be removed as a result of the operation applied ($k= i+1$ and $k+1=j$). However, Lemma 3.5 cannot remove a super hard breakpoint.

#### Lemma 3.6

*Given a valid unsigned instance* $I=(\pi ,\breve{\pi },\breve{\iota })$ *for the* ${\textsc {SbIRT}}$ *problem, if* $b_1(I) > 0$ *and there is no pair of softly connected breakpoints, then there must be at least one overcharged breakpoint.*

#### Proof

Assume that there are no overcharged breakpoints in *I*. We will show by contradiction that $\sum _{\breve{\pi }_i \in \breve{\pi }} \breve{\pi }_i < \sum _{\breve{\iota }_i \in \breve{\iota }} \breve{\iota }_i$, which contradicts the fact that *I* is a valid instance. Since there is no pair of softly connected breakpoints, it follows that for each soft breakpoint $(\pi _{i},\pi _{i+1})$, we have $\breve{\pi }_{i+1} < \breve{\iota }_{k}$, where $k = max(\pi _{i},\pi _{i+1})$, otherwise *I* has at least one pair of softly connected breakpoints.

Let ${\mathcal {S}}$ be the set of soft breakpoints from *I*. We have that $\sum _{(\pi _{i},\pi _{i+1}) \in {\mathcal {S}}} \breve{\pi }_{i+1} < \sum _{(\pi _{i},\pi _{i+1}) \in {\mathcal {S}}} \breve{\iota }_{max(\pi _{i},\pi _{i+1})}$, which means that there are not enough nucleotides in soft breakpoints to remove all of them while not turning them into undercharged breakpoints. Besides, for each undercharged breakpoint $(\pi _{i},\pi _{i+1})$ we also have that $\breve{\pi }_{i+1} < \breve{\iota }_{k}$, where $k = max(\pi _{i},\pi _{i+1})$. Since *I* has no overcharged breakpoints, it follows that $\sum _{\breve{\pi }_i \in \breve{\pi }} \breve{\pi }_i < \sum _{\breve{\iota }_i \in \breve{\iota }} \breve{\iota }_i$, and *I* is not a valid instance. $\square$

#### Lemma 3.7

*Given a valid unsigned instance* $I = (\pi ,\breve{\pi },\breve{\iota })$ *for the* ${\textsc {SbIRT}}$ *problem, if* *I* *has only one overcharged breakpoint* $(\pi _{i},\pi _{i+1})$*, one undercharged breakpoint* $(\pi _{j},\pi _{j+1})$*, and there is no pair of softly connected breakpoints, then* $\breve{\pi }_{i+1} + \breve{\pi }_{j+1} \ge \breve{\iota }_{x} + \breve{\iota }_{y}$*, where* $x = max(\pi _{i},\pi _{i+1})$ and $y = max(\pi _{j},\pi _{j+1})$.

#### Proof

By contradiction, assume that $\breve{\pi }_{i+1} + \breve{\pi }_{j+1} < \breve{\iota }_{x} + \breve{\iota }_{y}$. Since no pair of softly connected breakpoints exist in *I*, it follows that there are no soft breakpoints in $(\pi ,\breve{\pi },\breve{\iota })$ or there are not enough nucleotides in the soft breakpoints to remove them. In both cases, moving the excess of nucleotides from the overcharged breakpoint $(\pi _{i},\pi _{i+1})$ to the undercharged breakpoint $(\pi _{j},\pi _{j+1})$ is not enough to remove two breakpoints ($\breve{\pi }_{i+1} + \breve{\pi }_{j+1} < \breve{\iota }_{x} + \breve{\iota }_{y}$). So, the instance $(\pi ,\breve{\pi },\breve{\iota })$ remains with at least one undercharged breakpoint $(\pi _{j},\pi _{j+1})$ and possibly with soft breakpoints with not enough nucleotides to remove them, which contradicts the fact that $\sum _{\breve{\pi }_i \in \breve{\pi }} \breve{\pi }_i = \sum _{\breve{\iota }_i \in \breve{\iota }} \breve{\iota }_i$. $\square$

#### Lemma 3.8

*Given an unsigned instance* $I = (\pi ,\breve{\pi },\breve{\iota })$ *for the* ${\textsc {SbIRT}}$ *problem, if* *I* *has only one overcharged breakpoint* $(\pi _{i},\pi _{i+1})$*, at least one undercharged breakpoint* $(\pi _{j},\pi _{j+1})$*, and there is no pair of softly connected breakpoints, then there is a sequence of two operations that removes at least two breakpoints.*

#### Proof

By Lemma 3.7 we have that $\breve{\pi }_{i+1} + \breve{\pi }_{j+1} \ge \breve{\iota }_{x} + \breve{\iota }_{y}$, where $x = max(\pi _{i},\pi _{i+1})$ and $y = max(\pi _{j},\pi _{j+1})$. If $\breve{\pi }_{i+1} + \breve{\pi }_{j+1} = \breve{\iota }_{x} + \breve{\iota }_{y}$, assume without loss of generality that $i < j$. We apply a sequence of two reversals $\rho ^{(i+1,j)}_{(\breve{\iota }_x,0)}\cdot \rho ^{(i+1,j)}_{(\breve{\iota }_x,0)}$ to move the exceeding nucleotides from $\breve{\pi }_{i+1}$ to $\breve{\pi }_{j+1}$, and both breakpoints are removed.

If $\breve{\pi }_{i+1} + \breve{\pi }_{j+1} > \breve{\iota }_{x} + \breve{\iota }_{y}$, then at least a third breakpoint must exist. By Lemma 3.3, it is possible to redistribute the nucleotides within intergenic regions $\breve{\pi }_i$, $\breve{\pi }_j$, and $\breve{\pi }_k$ using two consecutive transpositions. Initially, we verify if there is a soft breakpoint to receive $(\breve{\iota }_{x} + \breve{\iota }_{y}) - (\breve{\pi }_{i+1} + \breve{\pi }_{j+1})$ nucleotides. Note that adding or removing nucleotides to a soft breakpoint does not turn it into a hard breakpoint. If the soft breakpoint exists, then the overcharged and undercharged breakpoints will be removed and it will receive the exceeding nucleotides after applying two consecutive transpositions. Otherwise, the third breakpoint must be an undercharged breakpoint, which can be removed or turned into an overcharged breakpoint after receiving the exceeding nucleotides.

In the worst case, two breakpoints are removed after applying a sequence of two operations, and the lemma follows. $\square$

Note that the sequence of operations from Lemma 3.8 generates at most one overcharged breakpoint after two consecutive transpositions, but if it occurs the instance $(\pi ,\breve{\pi },\breve{\iota })$ will have no soft breakpoints.

#### Lemma 3.9

*Given an unsigned instance* $I=(\pi ,\breve{\pi },\breve{\iota })$ *for the* ${\textsc {SbIRT}}$ *problem such that* $b_s(I) > 0$ *and with no pair of softly connected breakpoints, it is possible to create a hard undercharged breakpoint keeping the instance with no pair of softly connected breakpoints, or create a super hard undercharged breakpoint after applying one operation of reversal or transposition.*

#### Proof

If there is at least one decreasing strip in $\pi$, then must exist a pair of soft breakpoints $(\pi _{i},\pi _{i+1})$ and $(\pi _{j},\pi _{j+1})$, with $i <j$, such that $(\pi _{i},\pi _{j})$ or $(\pi _{i+1},\pi _{j+1})$ are consecutive in $\iota$ [19]. If $(\pi _{i},\pi _{j})$ are consecutive in $\iota$, then we apply a reversal $\rho _{(\breve{\pi }_{i+1},\breve{\pi }_{j+1})}^{(i+1, j)}$. Otherwise, we apply a reversal $\rho _{(0, 0)}^{(i+1, j)}$. Note that in both cases all the nucleotides are moved to the hard undercharged breakpoint created, which guarantees that the instance remains with no pair of softly connected breakpoints. If there is no decreasing strip in $\pi$, it is always possible to find three soft breakpoints $(\pi _{i},\pi _{i+1})$, $(\pi _{j},\pi _{j+1})$, and $(\pi _{k},\pi _{k+1})$, such that a transposition $\tau _{(0,0,0)}^{(i+1,j+1,k+1)}$ creates a hard undercharged breakpoint and no hard breakpoint is removed [7]. Besides, since the instance has only increasing strips, it guarantees that the hard undercharged breakpoint created (joining two increasing strips) is a super hard undercharged breakpoint, and the lemma follows. $\square$

#### Lemma 3.10

*Given an unsigned instance* $I=(\pi ,\breve{\pi },\breve{\iota })$ *for the* ${\textsc {SbIRT}}$ *problem such that there is only one overcharged breakpoint, no undercharged breakpoints, and there is no pair of softly connected breakpoints, then there is a sequence of at most three operations that removes at least two breakpoints or a sequence of at most four operations that removes at least three breakpoints.*

#### Proof

Note that $b_s(\pi ,\breve{\pi },\breve{\iota }) \ge 2$, since it is impossible to create a valid instance with only one overcharged breakpoint and one soft breakpoint. Applying Lemma 3.9 we have two possibilities: (i) a hard undercharged breakpoint is created keeping the instance with no pair of softly connected breakpoints, then Lemma 3.8 can be applied (resulting in two breakpoints removed after applying three operations); (ii) a super hard undercharged breakpoint is created. In this case, if there are no pair of softly connected breakpoints in *I*, then Lemma 3.8 can be applied (also resulting in two breakpoints removed after applying three operations). Otherwise, Lemma 3.5 can be applied. Note that, by Remark 3.1, the super hard undercharged breakpoint remains untouched, and one of the following cases can occur:

- A new overcharged breakpoint is created, and Lemma 3.4 can be applied (three breakpoints removed after applying four operations).
- A pair of softly connected breakpoints is created, and Lemma 3.5 can be applied (two breakpoints removed after applying three operations).
- There is no pair of softly connected breakpoints in *I*, and Lemma 3.8 can be applied (three breakpoints removed after applying four operations).

$$\square$$

#### Remark 3.2

Note that if only two breakpoints are removed by Lemma 3.10, then it implies that the resulting genome $(\pi ,\breve{\pi })$ is different from $(\iota ,\breve{\iota })$.

Now consider Algorithm 1, which consists of four cases depending on the number of overcharged breakpoints or the existence of a pair of softly connected breakpoints. 
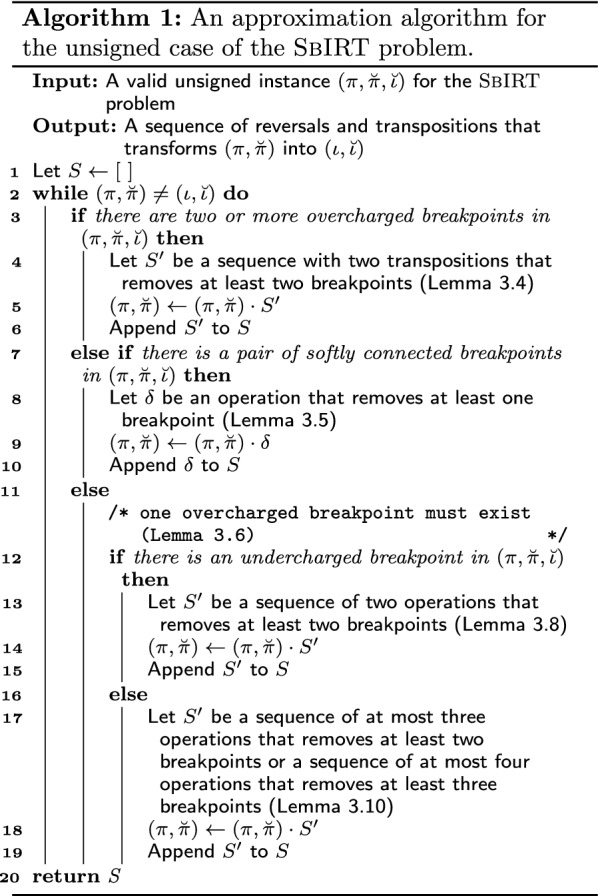


Note that at each iteration of Algorithm 1, at least one breakpoint is removed, so eventually $(\pi ,\breve{\pi })$ will be transformed into $(\iota ,\breve{\iota })$ and the algorithm stops. Besides, each step is performed in linear time using the auxiliary structures of a breakpoint list and the inverse permutation of $\pi$ (i.e., a permutation that indicates the position of each element *i* in $\pi$). Since $b(\pi ,\breve{\pi },\breve{\iota }) \le n + 1$, the running time of Algorithm 1 is ${\mathcal {O}}(n^2)$.

#### Lemma 3.11

*Given an unsigned instance* $I=(\pi ,\breve{\pi },\breve{\iota })$ *for the* ${\textsc {SbIRT}}$ *problem, Algorithm* 1 *transforms* $(\pi ,\breve{\pi })$ into $(\iota ,\breve{\iota })$ *using at most* $\frac{4b_1(\pi ,\breve{\pi },\breve{\iota })}{3}$ *operations.*

#### Proof

Algorithm 1 can be analyzed considering the following cases:

1. *I* has at least two overcharged breakpoints (lines 3 to 6).
2. *I* has at least one pair of softly connected breakpoints (lines 7 to 10).
3. *I* has only one overcharged breakpoint, at least one undercharged breakpoint, and there is no pair of softly connected breakpoints (lines 12 to 15).
4. *I* has only one overcharged breakpoint, no undercharged breakpoints, and there is no pair of softly connected breakpoints (lines 16 to 19).

Note that, if the algorithm reaches cases 3 or 4, there is exactly one overcharged breakpoint. Otherwise, case 1 would be performed first or the instance is not a valid one (Lemma 3.6).

Cases 1, 2, and 3 remove, on average, one breakpoint per operation. If the worst case of Case 4 is performed (where two breakpoints are removed with three operations), we have by Remark 3.2 that $(\pi ,\breve{\pi }) \ne (\iota ,\breve{\iota })$, and cases 1, 2, or 3 will be applied subsequently, and all guarantees a sequence of operations that will remove, on average, one breakpoint per operation. Thus, on average, each breakpoint is removed by using at most $\frac{4}{3}$ operations, and the lemma follows. $\square$

#### Theorem 3.1

*Algorithm* 1 *is a 4-approximation algorithm for the unsigned case of the* ${\textsc {SbIRT}}$ *problem.*

#### Proof

Given an unsigned instance $I = (\pi ,\breve{\pi },\breve{\iota })$ for the ${\textsc {SbIRT}}$ problem, we have by Proposition 3.1 that $d_{{\textsc {SbIRT}}}(I) \ge \frac{b_1(I)}{3}$. By Lemma 3.11, Algorithm 1 transforms $(\pi ,\breve{\pi })$ into $(\iota ,\breve{\iota })$ using at most $\frac{4b_1(\pi ,\breve{\pi },\breve{\iota })}{3}$ operations. Thus, we obtain the following approximation ratio:

$$\begin{aligned} \frac{\frac{4b_1(\pi ,\breve{\pi },\breve{\iota })}{3}}{\frac{b_1(\pi , \breve{\pi },\breve{\iota })}{3}} = \frac{12}{3} = 4. \end{aligned}$$

$$\square$$

#### Incorporating generic transpositions

In this section we use a more generalized definition of transpositions and design a 3-approximation algorithm for the sorting by intergenic reversals and transpositions problem using that definition. Let us start with a formal definition of intergenic moves and generic transpositions, that include intergenic transpositions and intergenic moves.

##### Definition 3.1

An intergenic move $\tau ^{(i,i,k)}_{(x,y,z)}$, with $1 \le i < k \le n+1$, $x \in [0..\breve{\pi }_i-1]$, $y \in [1..\breve{\pi }_i]$, $x < y$, and $z \in [0..\breve{\pi }_k]$ cuts $\breve{\pi }_i$ into three parts of sizes *x*, $y-x$ and $\breve{\pi }_i-y$, and cuts $\breve{\pi }_k$ after *z* nucleotides, and inserts the segment from $\breve{\pi }_i$ of size $y-x$ into $\breve{\pi }_k$ after the *z*-th nucleotide. This means that $(\pi ,\breve{\pi })\cdot \tau ^{(i,i,k)}_{(x,y,z)}$ results in $(\pi ,\breve{\pi }')$, with $\breve{\pi }'_j = \breve{\pi }_j$ if $j \not \in \{i,k\}$, $\breve{\pi }'_i = \breve{\pi }_i - (y - x)$, and $\breve{\pi }'_k = \breve{\pi }_k + (y - x)$.

Similarly, an intergenic move $\tau ^{(i,k,k)}_{(x,y,z)}$, with $1 \le i < k \le n+1$, $x \in [0..\breve{\pi }_i]$, $y \in [0..\breve{\pi }_k-1]$, $z \in [1..\breve{\pi }_k]$, and $y < z$ cuts $\breve{\pi }_i$ after *x* nucleotides, cuts $\breve{\pi }_k$ into three parts of sizes *y*, $z-y$ and $\breve{\pi }_k-z$ nucleotides, and inserts the segment from $\breve{\pi }_k$ of size $z-y$ into $\breve{\pi }_i$ after the *x*-th nucleotide. This means that $(\pi ,\breve{\pi })\cdot \rho$ results in $(\pi ,\breve{\pi }')$, with $\breve{\pi }'_j = \breve{\pi }_j$ if $j \not \in \{i,k\}$, $\breve{\pi }'_i = \breve{\pi }_i + z - y$, and $\breve{\pi }'_k = \breve{\pi }_k - (z - y)$.

##### Definition 3.2

A generic transposition $\tau ^{(i,j,k)}_{(x,y,z)}$, with $1 \le i < k \le n+1$, is an intergenic move (as in Definition 3.1), if $i = j$ or $j=k$, or is an intergenic transposition (as in Definition 2.2), where $1 \le i< j < k \le n+1$.

We note that an intergenic move modifies only two intergenic regions of an instance. Now we show how generic transpositions affect the number of breakpoints from an instance $(\pi ,\breve{\pi },\breve{\iota })$.

##### Lemma 3.12

*Given an unsigned instance* $I_1=(\pi , \breve{\pi },\breve{\iota })$ *and a signed instance* $I_2=(\pi , \breve{\pi },\breve{\iota })$ *of the* ${\textsc {SbIRT}}$ *problem,* $\Delta b_1(I_1,\tau ) \ge -3$ *and* $\Delta b_2(I_2,\tau ) \ge -3$ *for any generic transposition* $\tau ^{(i,j,k)}_{(x,y,z)}$*, respectively.*

##### Proof

The proof is similar to the one described in Lemma 3.1, and considering that an intergenic transposition can affect up to three breakpoints and an intergenic move can affect up to two breakpoints. $\square$

In the following lemma we explain how to remove an overcharged breakpoint using one intergenic move.

##### Lemma 3.13

*Given an unsigned instance* $I = (\pi ,\breve{\pi },\breve{\iota })$ *for the* ${\textsc {SbIRT}}$ *problem, if* *I* *has one overcharged breakpoint, then it is possible to remove at least one breakpoint using an intergenic move.*

##### Proof

**Fig. 4:**
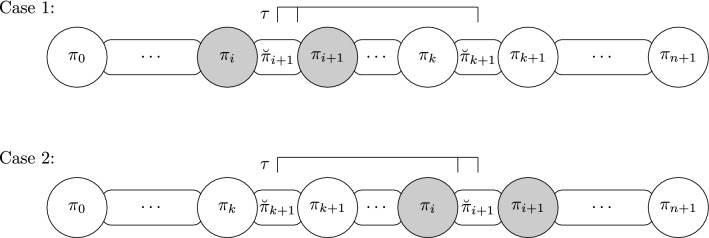
Illustration of an intergenic move applied to remove an overcharged breakpoint. The overcharged breakpoint is represented with a grayscale color. On the top (Case 1), the intergenic move $\tau _{(x,y,z)}^{(i+1,i+1,k+1)}$ is applied to move the excess of nucleotides from the overcharged breakpoint $(\pi _{i},\pi _{i + 1})$ to the breakpoint $(\pi _{k},\pi _{k + 1})$, such that $i < k$. Similarly, at the bottom (Case 2), the intergenic move $\tau _{(x,y,z)}^{(k+1,i+1,i+1)}$ is applied but $i > k$

Let $(\pi _{i},\pi _{i + 1})$, with $0 \le i \le n$, be the overcharged breakpoint, and let $w = \breve{\iota }_{x} - \breve{\pi }_{i + 1}$, such that $x = \max (\pi _{i},\pi _{i + 1})$. We note that another breakpoint $(\pi _{k},\pi _{k + 1})$, with $0 \le k \le n$ and $k \ne i$, must exist in $(\pi ,\breve{\pi },\breve{\iota })$, otherwise the instance is not valid. We can use an intergenic move to transfer *w* nucleotides from $\breve{\pi }_{i + 1}$ to $\breve{\pi }_{k + 1}$, and the overcharged breakpoint is removed. If $i < k$, we can apply the intergenic move $\tau ^{(i + 1,i + 1,k)}_{(0,w,0)}$ (Fig. 4, Case 1); otherwise we can apply the intergenic move $\tau ^{(k + 1,i + 1,i + 1)}_{(0,0,w)}$(Fig. 4, Case 2). $\square$

##### Lemma 3.14

*Given a valid unsigned instance* $I=(\pi ,\breve{\pi },\breve{\iota })$ *for the* ${\textsc {SbIRT}}$ *problem, if* $b(I) > 0$ *and there are no overcharged breakpoints, then there must be at least one pair of softly connected breakpoints.*

##### Proof

Note that, since there is no overcharged breakpoint, and $b(I) > 0$, then at least two soft breakpoints must exist, otherwise the instance has only undercharged breakpoints and it is not valid. We can use a similar argument as the proof of Lemma 3.6 to show that at least one pair of soft breakpoints must be connected, otherwise *I* is not a valid instance. $\square$

Algorithm 2 consists of two cases: one occurs when there is an overcharged breakpoint and the other is applied when there are only soft and undercharged breakpoints. At each iteration of Algorithm 2 at least one breakpoint is removed using one reversal or one generic transposition, so eventually $(\pi ,\breve{\pi })$ will be transformed into $(\iota ,\breve{\iota })$ and the algorithm ends. The same argument of Algorithm 1 can be used to show that the running time of Algorithm 2, which is ${\mathcal {O}}(n^2)$. 
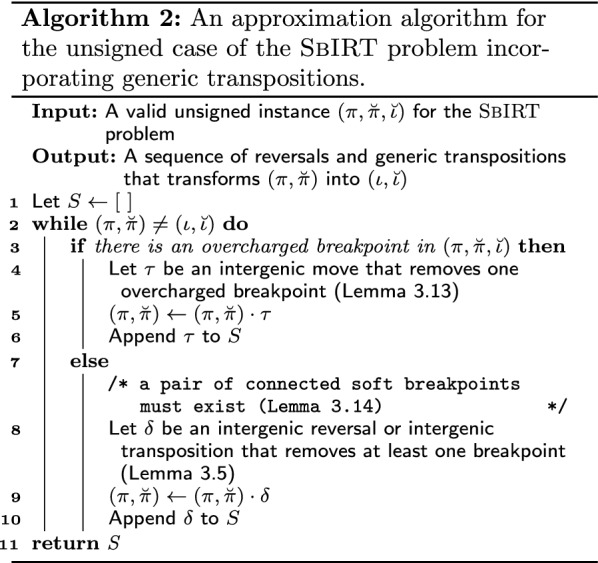


##### Lemma 3.15

*Given an unsigned instance* $I=(\pi ,\breve{\pi },\breve{\iota })$ *for the* ${\textsc {SbIRT}}$ *problem, Algorithm* 2 *transforms* $(\pi ,\breve{\pi })$ *into* $(\iota ,\breve{\iota })$ *using at most* $b_1(\pi ,\breve{\pi },\breve{\iota })$ *operations.*

##### Proof

Algorithm 2 has only two cases: (i) *I* has at least one overcharged breakpoint (lines 3 to 6) and (ii) *I* has at least one pair of softly connected breakpoints (lines 7 to 10). In both cases at least one breakpoint is removed per operation, and the lemma follows. $\square$

##### Theorem 3.2

*Algorithm* 2 *is a 3-approximation algorithm for the unsigned case of* ${\textsc {SbIRT}}$ *problem incorporating generic transpositions.*

##### Proof

Since Lemma 3.12 has the same lower bound for $\Delta b_1(\pi , \breve{\pi },\breve{\iota },\tau )$ as in Lemma 3.2, Proposition 3.1 is also valid when considering intergenic reversals and generic transpositions. Given an unsigned instance $I = (\pi ,\breve{\pi },\breve{\iota })$ for the ${\textsc {SbIRT}}$ problem, we have by Proposition 3.1 that $d_{{\textsc {SbIRT}}}(I) \ge \frac{b_1(I)}{3}$. By Lemma 3.15, Algorithm 2 transforms $(\pi ,\breve{\pi })$ into $(\iota ,\breve{\iota })$ using at most $b_1(\pi ,\breve{\pi },\breve{\iota })$ operations, and the lemma follows. $\square$

#### Greedy strategy

To improve the practical performance of algorithms 1 and 2, we search at the beginning of each iteration for one of the following operations.

1. A transposition that removes three breakpoints.
2. A reversal or transposition that removes two breakpoints.

The search is performed in linear time knowing where each element is placed in $\pi$. Therefore, it does not increase the asymptotic time complexity of algorithms 1 and 2. Besides, this strategy does not affect the theoretical approximation factors of algorithms 1 and 2, since the applied operations remove at least two breakpoints each.

### Approximation algorithms for the signed case of the ${\textsc {SbIRT}}$ problem

In this section, we show how to obtain approximation algorithms for the signed case of the ${\textsc {SbIRT}}$ problem based on a reduction from a signed instance into an unsigned instance.

The algorithms are designed following three steps: (i) Initially, we describe a polynomial time function ${\mathcal {F}}$ that maps a signed instance $I=(\pi ,\breve{\pi },\breve{\iota })$ of the ${\textsc {SbIRT}}$ problem into a valid unsigned instance $I^\prime =(\pi ^\prime ,\breve{\pi }^\prime ,\breve{\iota }^\prime )$. (ii) Then, we use the algorithms 1 or 2 to provide an solution $S(I^\prime )$ for the instance $I^\prime$, and (iii) we show a polynomial time function ${\mathcal {G}}$ that maps a solution $S(I^\prime )$ into a valid solution *S*(*I*) for *I*. Lastly, we prove the theoretical approximation factor obtained by adopting this process.

Function ${\mathcal {F}}$ works as follows: for each element $\pi _i$ of the source genome $(\pi ,\breve{\pi })$, we map it into two new elements: $(2\pi _i - 1, 2\pi _i$), if $\pi _i > 0$, and $(2|\pi _i|, 2|\pi _i| - 1)$, otherwise. In both cases, a new intergenic region with size zero is inserted between these two new elements. We apply the same procedure in the target genome $(\iota , \breve{\iota })$. This procedure doubles the size of the instance $I^\prime$ but note that $b_2(I) = b_1(I^\prime )$, since each breakpoint type two is mapped into a breakpoint type one. Besides, the ${\mathcal {F}}$ function takes linear time to complete the mapping.

Function ${\mathcal {G}}$ uses the fact that algorithms 1 and 2 act only over breakpoints to map a solution $S(I^\prime )$ for $I^\prime$ into a valid solution *S*(*I*) for *I*. It maps each reversal $\rho ^{(i,j)}_{(x,y)}$ in $S(I^\prime )$ into $\rho ^{(i^\prime ,j^\prime )}_{(x,y)}$ such that $i^\prime =\frac{i + 1}{2}$ and $j^\prime =\frac{j}{2}$, and each transposition (or generic transposition) $\tau ^{(i,j,k)}_{(x,y,z)}$ in $S(I^\prime )$ into $\tau ^{(i^\prime ,j^\prime ,k^\prime )}_{(x,y,z)}$ such that $i^\prime =\frac{i + 1}{2}$, $j^\prime = \frac{j + 1}{2}$, and $k^\prime =\frac{k + 1}{2}$. Recall that this mapping is only possible because algorithms 1 and 2 do not create breakpoints of type one during the process that transform the source genome into the target genome. Furthermore, note that solutions *S*(*I*) and $S(I^\prime )$ have the same number of operations. Since solution $S(I^\prime )$ is ${\mathcal {O}}(n)$, where *n* is the number of elements of $\pi$, then function ${\mathcal {G}}$ takes linear time to complete the solution mapping.

**Fig. 5:**
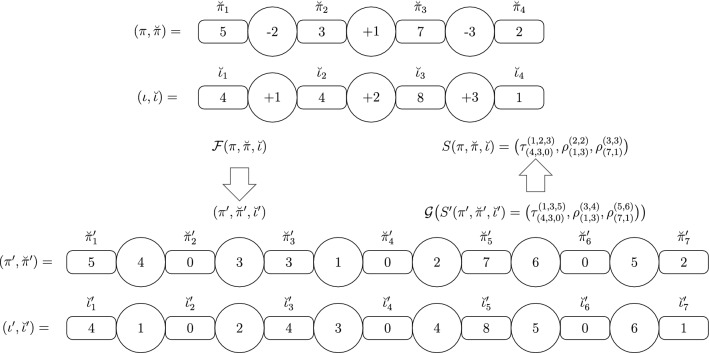
On top, we have a signed instance $(\pi ,\breve{\pi },\breve{\iota })$ of the ${\textsc {SbIRT}}$ problem, with $\pi =({-2}~{+1}~{-3})$, $\breve{\pi }=(5,3,7,2)$, and $\breve{\iota }=(4,4,8,1)$, which is mapped by the ${\mathcal {F}}$ function into an unsigned instance $(\pi ^{\prime },\breve{\pi }^{\prime },\breve{\iota }^{\prime })$ (at the bottom), such that $\pi ^{\prime }=(4~3~1~2~6~5)$, $\breve{\pi }^{\prime }=(5,0,3,0,7,0,2)$, and $\breve{\iota }^{\prime }=(4,0,4,0,8,0,1)$. The ${\mathcal {G}}$ function maps a solution $S^{\prime }=\big (\tau ^{(1,3,5)}_{(4,3,0)},\rho ^{(3,4)}_{(1,3)},\rho ^{(5,6)}_{(7,1)}\big )$ for the instance $(\pi ^{\prime },\breve{\pi }^{\prime },\breve{\iota }^{\prime })$ into a valid solution $S=\big (\tau ^{(1,2,3)}_{(4,3,0)},\rho ^{(2,2)}_{(1,3)},\rho ^{(3,3)}_{(7,1)}\big )$, with same size, for the instance $(\pi ,\breve{\pi },\breve{\iota })$

Figure 5 shows an example using the functions ${\mathcal {F}}$ and ${\mathcal {G}}$. The signed instance $(\pi ,\breve{\pi },\breve{\iota })$ of the ${\textsc {SbIRT}}$ problem (at the top) is mapped into an unsigned instance $(\pi ^{\prime },\breve{\pi }^{\prime },\breve{\iota }^{\prime })$ (at the bottom) using the function ${\mathcal {F}}$. Moreover, the function ${\mathcal {G}}$ is used to map an solution $S^{\prime }$ for $(\pi ^{\prime },\breve{\pi }^{\prime },\breve{\iota }^{\prime })$ into a valid solution *S* of same size for $(\pi ,\breve{\pi },\breve{\iota })$.

Algorithms 3 and 4 show the steps to obtain a solution for the signed case of the ${\textsc {SbIRT}}$ problem. 
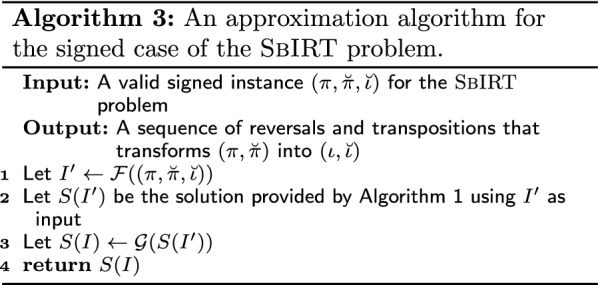

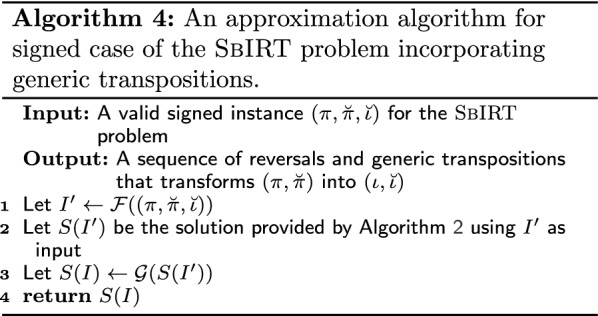




Note that the functions ${\mathcal {F}}$ and ${\mathcal {G}}$ take linear time. Thus, the running time of algorithms 3 and 4 are ${\mathcal {O}}(n^2)$. Now we show that algorithms 3 and 4 guarantee the approximation factors of 4 and 3 considering the reversal and transposition events and incorporating the generic transposition, respectively.

#### Lemma 3.16

*Given a signed instance* $I=(\pi ,\breve{\pi },\breve{\iota })$ *for the* ${\textsc {SbIRT}}$ *problem, we have that* $b_2(I) = b_1(I^\prime )$*, where* $I^\prime = {\mathcal {F}}(I)$.

#### Proof

Direct by the construction of the ${\mathcal {F}}$ function. $\square$

#### Lemma 3.17

*Given a signed instance* $I=(\pi ,\breve{\pi },\breve{\iota })$ *for the* ${\textsc {SbIRT}}$ *problem, Algorithm* 3 *transforms* $(\pi ,\breve{\pi })$ *into* $(\iota , \breve{\iota })$ *using up to* $\frac{4b_2(\pi ,\breve{\pi },\breve{\iota })}{3}$ *reversals and transpositions.*

#### Proof

By Lemma 3.16, we have that $b_2(I) = b_1(I^\prime )$, where $I^\prime = {\mathcal {F}}(I)$. Besides, a solution $S(I^\prime )$ for $I^\prime$ is obtained using up to $\frac{4b_1(\pi ,\breve{\pi },\breve{\iota })}{3}$ reversals and transpositions. Since a valid solution *S*(*I*) for *I* generated by the ${\mathcal {G}}$ function has the same size as $S(I^\prime )$, the lemma follows. $\square$

#### Theorem 3.3

*Algorithm* 3 *is a 4-approximation algorithm for the signed case of the* ${\textsc {SbIRT}}$ *problem.*

#### Proof

Given an signed instance $I = (\pi ,\breve{\pi },\breve{\iota })$ for the ${\textsc {SbIRT}}$ problem, we have by Proposition 3.2 that $d_{{\textsc {SbI}}\overline{\text {R}}{\textsc {T}}{}}(I) \ge \frac{b_2(I)}{3}$. By Lemma 3.17, we have that Algorithm 3 transforms $(\pi ,\breve{\pi })$ into $(\iota ,\breve{\iota })$ using at most $\frac{4b_2(\pi ,\breve{\pi },\breve{\iota })}{3}$ operations of reversal and transposition, and the theorem follows. $\square$

#### Lemma 3.18

*Given a signed instance* $I=(\pi ,\breve{\pi },\breve{\iota })$ *for the* ${\textsc {SbIRT}}$ *problem, Algorithm* 4 *transform* $(\pi ,\breve{\pi })$ *into* $(\iota , \breve{\iota })$ *using at most* $b_2(\pi ,\breve{\pi },\breve{\iota })$ *operations of reversal and transposition.*

#### Proof

The proof is similar to the one described in Lemma 3.17 but considering that a solution $S(I^\prime )$ for the instance $I^\prime$ is obtained using at most $b_1(\pi ,\breve{\pi },\breve{\iota })$ operations of reversal and transposition. $\square$

#### Theorem 3.4

*Algorithm* 4 *is a 3-approximation algorithm for the signed case of the* ${\textsc {SbIRT}}$ *problem incorporating generic transpositions*.

#### Proof

Given an signed instance $I = (\pi ,\breve{\pi },\breve{\iota })$ for the ${\textsc {SbIRT}}$ problem, we have by lemmas 3.1, 3.2, and 3.12 the following lower bound: $d_{{\textsc {SbI}}\overline{\text {R}}{\textsc {T}}{}}(I) \ge \frac{b_2(I)}{3}$. By Lemma 3.18, we have that Algorithm 4 transforms $(\pi ,\breve{\pi })$ into $(\iota ,\breve{\iota })$ using at most $b_2(\pi ,\breve{\pi },\breve{\iota })$ operations of reversal and transposition, and the theorem follows. $\square$

## Practical results

In this section, we compare the proposed algorithms using simulated datasets. Besides, we perform an experiment using marine and brackish picocyanobacteria genomes from Cyanorak 2.1 [20] system.

### Results with unsigned simulated datasets

To assess algorithms 1 and 2, we compare them with the 4.5-approximation algorithm for the unsigned case of the ${\textsc {SbIRT}}$ problem presented by Brito et. al. [16]. We hereafter refer to the 4.5-approximation algorithm [16], Algorithm 1, and Algorithm 2 by 4.5${\textsc {SbIRT}}$, 4${\textsc {SbIRT}}$, and 3SbIRGT, respectively. We used the following datasets of simulated genomes:

- DS1: This dataset was presented by Brito et. al. [16]. It is divided into groups according to the number of random operations (reversal or transposition) used to create each instance in the dataset. Each group contains 10.000 instances of size 100. Instances are created as follows: the target genome is composed by the identity permutation $\iota$ and the intergenic region sizes in the target genome are randomly chosen in the range [0..100]. The source genome was obtained after applying a sequence of random operations in the target genome. The number of random operations ranged from 5 up to 100, in intervals of 5. Reversals and transpositions can be selected with the same probability to create each instance. This dataset has a total of 200.000 instances.
- DS2: This dataset contains groups of instances with sizes 100, 200, 300, 400, and 500. Each group contains 10.000 instances. Instances are created as follows: the target genome is again composed by the identity permutation $\iota$ with intergenic region sizes randomly chosen in the range [0..100]. The source genome $(\pi , \breve{\pi })$ was obtained by shuffling the lists of genes and intergenic region sizes from the target genome independently, in order to create instances with a large number of breakpoints. This dataset has a total of 50.000 instances.

The DS1 dataset explores scenarios considering instances of same size and where the number of breakpoints tends to increase as the number of random operations used to generate each instance grows. The DS2 dataset explores scenarios considering groups of instances with different sizes and, by the random process of construction, they tend to have a higher number of breakpoints.

Tables 1, 2, and 3 consider the DS1 dataset and they use, respectively, algorithms 4.5${\textsc {SbIRT}}$, 4${\textsc {SbIRT}}$, and 3SbIRT. Columns OP, Default Implementation, and Greedy Strategy represent the number of random operations used to create the instances, the result with no greedy strategy, and the result with the greedy strategy, respectively.

From Table 1, we note that the greedy strategy significantly improved the results of the 4.5${\textsc {SbIRT}}$ algorithm. The minimum, average, and maximum metrics for the distance and the approximation ratio using the greedy strategy presented lower values when compared with the algorithm default implementation, except for the minimum distance when OP $=05$. The average approximation ratio tends to increase as OP increases. When no greedy strategy is applied, the values ranged from 2.01 (OP $=05$) to 2.96 (OP $=100$). Using the greedy strategy the values ranged from 1.34 (OP $=05$) to 2.11 (OP $=100$). Besides, by adopting the greedy strategy we were able to find at least one optimal solution in the groups where OP $=05$ and OP $=10$, indicated by the minimum approximation ratio column with value 1.00.

Table 2 shows a similar behavior for 4${\textsc {SbIRT}}$ regarding the increase of the average approximation ratio as OP grows, and the improvement obtained by the greedy strategy. Using no greedy strategy, the average distance of 4${\textsc {SbIRT}}$ is better than the average distance of 4.5${\textsc {SbIRT}}$ algorithm when the number of random operations (OP) is greater than or equals to 50. It indicates that the default implementation of the 4${\textsc {SbIRT}}$ algorithm tends to provide better results when the instance has many breakpoints. When we compare both algorithms using the greedy strategy, the 4${\textsc {SbIRT}}$ algorithm provides better results for the vast majority of the groups and metrics. Considering all groups and using the greedy strategy, the maximum approximation ratio obtained by both algorithms (4.5${\textsc {SbIRT}}$ and 4${\textsc {SbIRT}}$) was 3.00, which is considerably less than the theoretical approximation factor proven for them.

Table 3 shows that 3SbIRGT provided results similar to those presented by 4${\textsc {SbIRT}}$. Considering the average distance and average approximation ratio columns, we can see a slight improvement for all values of OP compared with the practical results of 4${\textsc {SbIRT}}$. This fact results from the inclusion of the intergenic move operation, which can reduce the number of operations needed to transform a genome into another. Besides, considering the versions without and with the greedy strategy, respectively, the maximum approximation ratios regarding all groups were 2.97 and 2.83. Using the greedy strategy, the average approximation ratio of 3SbIRGT ranged from 1.29 to 2.05, which is significantly less than the theoretical approximation factor.

Table 4 shows the results for the DS2 dataset using 4.5${\textsc {SbIRT}}$, 4${\textsc {SbIRT}}$, and 3SbIRGT. The average distances of the algorithms without greedy strategy were close to the instance sizes in all groups. Computing the absolute difference between the average distance and the instance sizes, the highest values provided by the 4.5${\textsc {SbIRT}}$, 4${\textsc {SbIRT}}$, and 3SbIRGT algorithms were 4.00 (Size=500), 0.42 (Size=500), and 0.08 (Size=100), respectively. The greedy strategy also led to important improvement of the results for all the algorithms and groups. With and without greedy strategy, the best results were provided by 3SbIRGT followed by the 4${\textsc {SbIRT}}$ and 4.5${\textsc {SbIRT}}$ algorithms regarding the average distance and average approximation ratio metrics.

Table 5 shows the average running time, in seconds, of the 4.5${\textsc {SbIRT}}$, 4${\textsc {SbIRT}}$, and 3SbIRGT algorithms per instance, comparing the default implementation (DI) and the greedy strategy (GS) using the DS2 dataset. Note that the greedy strategy is more time-consuming than the default implementation. The maximum average running time of an algorithm without greedy strategy was less than 0.20 seconds, while using the greedy strategy it was 0.65 seconds. Observing the improvement in the results given by the greedy strategy in Table 4, we highlight that the additional running time is a good trade-off regarding running time and solution quality.

Based on the results, the practical approximation ratio provided by the algorithms tends to be better than the theoretical approximation factors. Besides, it is noteworthy that the greedy strategy has brought a significant improvement on both datasets. Since incorporating this strategy does not change the asymptotic time complexity nor the theoretical approximation of the algorithms, it becomes an excellent alternative to obtain better results.

### Results with signed simulated datasets

To assess algorithms 3 and 4, we compare them with the 3-approximation and the 2.5-approximation algorithms for the signed case of the ${\textsc {SbIRT}}$ problem, respectively, which were presented by Oliveira *et. al.* [17]. We hereafter refer to the 3-approximation algorithm [17], 2.5-approximation algorithm [17], Algorithm 3, and Algorithm 4 by 3${\textsc {SbI}}\overline{\text {R}}{\textsc {T}}{}$, 2.5${\textsc {SbI}}\overline{\text {R}}{\textsc {GT}}$, 4${\textsc {SbI}}\overline{\text {R}}{\textsc {T}}{}$, and 3${\textsc {SbI}}\overline{\text {R}}{\textsc {GT}}$, respectively. The results of the 4${\textsc {SbI}}\overline{\text {R}}{\textsc {T}}{}$ and 3${\textsc {SbI}}\overline{\text {R}}{\textsc {GT}}$ algorithms were obtained adopting the greedy strategy. We used the $\hbox {DB}_{{\mathrm{SIRIT}}}$ and $\hbox {DB}_{{\mathrm{SIRGT}}}$ datasets presented by Oliveira et. al. [17], and they have the following characteristics: Each dataset started with 100 target genomes $(\iota ,\breve{\iota })$, such that $\iota$ has 100 elements, and each value of $\breve{\iota }_i$, with $1 \le i \le 101$, being chosen randomly and uniformly in interval [0..100]. After that, from each source genome $(\iota ,\breve{\iota })$ were generated 100 instances $(\pi ,\breve{\pi },\breve{\iota })$ by applying:

- DB_SIRIT_: *d* random operations of reversals and transpositions (being 50% of each) in each source genome $(\iota ,\breve{\iota })$.
- DB_SIRGT_: *d* random operations of reversals and generic transpositions (being 50% of reversals, 40% of transpositions, and 10% of moves) in each source genome $(\iota ,\breve{\iota })$.

The parameters of each applied operation were randomly generated considering the range of valid values. The value of *d* ranged from 10 up to 100, in intervals of 10. For each value of *d*, a group with 10,000 instances was generated. DB_SIRIT_ and DB_SIRGT_ datasets have a total of 100,000 instances each.

Tables 6 and 7 show the practical results of the algorithms using the DB_SIRIT_ and DB_SIRGT_ datasets, respectively. The approximation ratio for each instance was computed using the lower bound based on the weighted cycle graph structure [17, Theorems 3.8 and 7.6].

Table 6 compares the results obtained by the $3{\textsc {SbI}}\overline{\text {R}}{\textsc {T}}{}$ and $4{\textsc {SbI}}\overline{\text {R}}{\textsc {T}}{}$ algorithms. The columns Small and Small or Equal indicate, for each group, the percentage of solutions provided by the $4{\textsc {SbI}}\overline{\text {R}}{\textsc {T}}{}$ algorithm with strictly smaller size and with small or equal size, respectively, when compared to the solutions provided by the $3{\textsc {SbI}}\overline{\text {R}}{\textsc {T}}{}$ algorithm.

From Table 6, it is possible to observe that the $4{\textsc {SbI}}\overline{\text {R}}{\textsc {T}}{}$ algorithm, in all the groups, was able to provide better results considering the metrics of average approximation ratio and average distance. Besides, considering the groups with *d* greater than 20, the algorithm provided better solutions in more than 75% of the instances (column Small). Considering the groups with *d* greater than 30, the $4{\textsc {SbI}}\overline{\text {R}}{\textsc {T}}{}$ algorithm provided better or equivalent size solutions (column Small or Equal) in more than 96% of the instances. It is important to note that, as the value of *d* increases, the absolute difference between the average distance provided by the $3{\textsc {SbI}}\overline{\text {R}}{\textsc {T}}{}$ and $4{\textsc {SbI}}\overline{\text {R}}{\textsc {T}}{}$ algorithms also increases significantly. When *d* is greater than 50, the absolute difference between the average distances is superior to 10, which indicates that the $4{\textsc {SbI}}\overline{\text {R}}{\textsc {T}}{}$ algorithm tends to provide better solutions in scenarios where a higher number of operations were used.

Table 7 compares the results obtained by the 2.5${\textsc {SbI}}\overline{\text {R}}{\textsc {GT}}$ and 3${\textsc {SbI}}\overline{\text {R}}{\textsc {GT}}$ algorithms. The columns Small and Small or Equal indicate, for each group, the percentage of solutions provided by the 3${\textsc {SbI}}\overline{\text {R}}{\textsc {GT}}$ algorithm with strictly smaller size and with small or equal size, respectively, when compared to the solutions provided by the 2.5${\textsc {SbI}}\overline{\text {R}}{\textsc {GT}}$ algorithm.

From Table 7, we can note that the 2.5${\textsc {SbI}}\overline{\text {R}}{\textsc {GT}}$ algorithm, when compared to the 3SbIRGT algorithm, showed a slightly better result regarding the average approximation ratio and distance in the groups with $d=10$ and $d=20$. Considering these two groups ($d=10$ and $d=20$), the absolute difference between the average distance provided by the algorithms was less than 0.61. Besides, by column Small, we can notice that in the groups $d=10$ and $d=20$ the 3${\textsc {SbI}}\overline{\text {R}}{\textsc {GT}}$ algorithm provided better solutions in 32.30% and 34.77% of the instances, respectively. This shows that the 3${\textsc {SbI}}\overline{\text {R}}{\textsc {GT}}$ algorithm can act in a complementary way with the 2.5${\textsc {SbI}}\overline{\text {R}}{\textsc {GT}}$ algorithm, even in the cases where both provide similar results. Since better estimates tend to outcome in enhanced analysis, selecting the better result between each algorithm is a good alternative to assist in this task. Regarding the groups where *d* is greater than 20, the 3${\textsc {SbI}}\overline{\text {R}}{\textsc {GT}}$ algorithm provided better results considering the average approximation ratio and distance. Furthermore, in the same groups, the 3${\textsc {SbI}}\overline{\text {R}}{\textsc {GT}}$ algorithm provided better or equivalent size solutions (column Small or Equal) in more than 73% of the instances.

From Tables 6 and 7, it is possible to note that $4{\textsc {SbI}}\overline{\text {R}}{\textsc {T}}{}$ and 3${\textsc {SbI}}\overline{\text {R}}{\textsc {GT}}$ algorithms are robust and tend to provide practical results better than the theoretical bounds.

### Results with real genomes

To assess the 3${\textsc {SbI}}\overline{\text {R}}{\textsc {GT}}$ algorithm and analyze the behavior with real genomes, we compared it with the 2-approximation algorithm for the problem considering reversals and transpositions on signed permutations (ignoring the intergenic regions), presented by Walter et al. [7]. We hereafter refer to the 2-approximation algorithm [7] by 2sb$\overline{\text {R}}$ t. We used 97 genomes from Cyanorak 2.1 [20], which is a system for the visualization and curation of marine and brackish picocyanobacteria genomes. The system encompasses 51 synechococcus, 3 cyanobium, 41 prochlorococcus genomes, and 2 prochlorococcus metagenome-assembled genomes. For each genome, the number of genes ranged from 1834 to 4391, and replicated genes correspond to less than 5% of the total genes, on average.

We performed a preprocessing stage to ensure that the data fits the model constraints, which is divided in two steps:

1. Map the sequence of genes and the intergenic regions into $(\pi ,\breve{\pi })$: For each genome, we mapped the first occurrence of the genes into a permutation $\pi$ and computed the size of the intergenic regions to obtain $\breve{\pi }$.
2. Pairing: For each pair of genomes, we performed a pairing so that the genes and conserved blocks shared by both genomes were kept while the remaining genes were removed through a process that simulates a sequence of deletions.

After the preprocessing stage, we obtained for each pairing an instance $(\pi ,\breve{\pi },\breve{\iota })$. Note that the 2SbRT algorithm requires as input only the permutation $\pi$, since it was not designed to consider the intergenic regions.

Finally, 3${\textsc {SbI}}\overline{\text {R}}{\textsc {GT}}$ with the greedy strategy and the 2Sb$\overline{\text {R}}$ t were applied to each pairing. The number of genome rearrangement events for each pairing was computed by the total of deletions used in the preprocessing stage (step 2) plus the size of the sequence of reversals and (generic) transpositions provided by the algorithms. These numbers were fed into a matrix of pairwise distances.

We constructed two phylogenetic trees based on the matrix of pairwise distances computed from the algorithms and using the Circular Order Reconstruction method [21]. To analyze the topological characteristics of the phylogenetic trees, we performed a comparison with the phylogenetic tree presented by Laurence et al. [20] using a tool [22] based on the maximum agreement subtrees (MAST) to determine the topological congruence between two phylogenetic trees. Table 8 shows the obtained results.

**Fig. 6:**
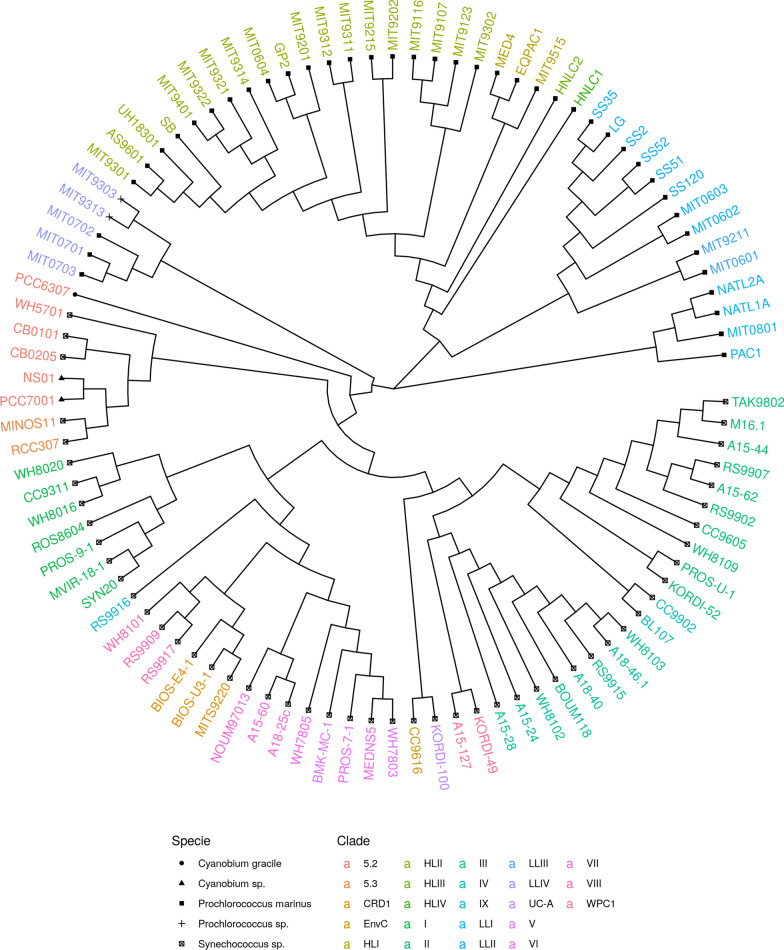
Phylogenetic tree based on genome rearrangements using the 3${\textsc {SbI}}\overline{\text {R}}{\textsc {GT}}$ algorithm with the greedy strategy and 97 genomes from the Cyanorak 2.1 system

Table 8 indicates that both phylogenetic tree have a high concordance with the phylogenetic trees presented by Laurence et al. [20], with the phylogenetic tree obtained from the 3${\textsc {SbI}}\overline{\text {R}}{\textsc {GT}}$ algorithm providing a MAST with more leaves and consequently a better value for $I_{cong}$ and P-value. It is important to mention that the objective of this experiment using real genomes is to demonstrate the applicability of our algorithm, which considers the information regarding the genes and the size of the intergenic regions, compared with a similar model that considers only the order and orientation of the genes. We used the same data preprocessing stage and reconstruction method to provide a fair comparison. However, the results may differ especially considering genomes with different characteristics and the adopted reconstruction method. Figure 6 shows a phylogenetic tree constructed using the Circular Order Reconstruction method [21] with the matrix of pairwise distances from the 3${\textsc {SbI}}\overline{\text {R}}{\textsc {GT}}$ algorithm.

**Table 1:** Comparison between the default implementation and the greedy strategy of the 4.5${\textsc {SbIRT}}$ algorithm using the DS1 dataset

OP	Default implementation						Greedy strategy					
	Distance			Approx. ratio			Distance			Approx. ratio		
	Min.	Avg.	Max.	Min.	Avg.	Max.	Min.	Avg.	Max.	Min.	Avg.	Max.
05	4	8.64	15	1.25	2.01	3.00	4	5.67	14	1.00	1.34	3.00
10	10	17.54	26	1.50	2.25	3.00	8	13.09	25	1.00	1.69	3.00
15	17	26.72	38	1.73	2.46	3.00	13	20.80	33	1.15	1.91	3.00
20	25	35.72	47	1.86	2.63	3.08	16	27.67	40	1.33	2.03	2.85
25	33	43.81	56	2.06	2.74	3.07	23	33.45	45	1.39	2.09	2.79
30	39	50.97	66	2.32	2.81	3.07	28	38.50	53	1.58	2.12	2.79
35	43	57.06	70	2.50	2.86	3.06	30	42.74	57	1.58	2.14	2.82
40	49	62.43	77	2.59	2.88	3.10	34	46.40	60	1.64	2.14	2.68
45	54	67.11	80	2.62	2.90	3.09	39	49.66	63	1.71	2.15	2.76
50	59	71.14	83	2.67	2.92	3.09	39	52.25	69	1.69	2.14	2.76
55	60	74.69	89	2.73	2.93	3.08	42	54.70	69	1.65	2.14	2.67
60	64	77.80	91	2.72	2.94	3.08	44	56.75	70	1.74	2.14	2.62
65	68	80.50	93	2.70	2.94	3.08	44	58.51	73	1.74	2.13	2.59
70	71	82.89	95	2.79	2.94	3.10	48	60.06	74	1.73	2.13	2.57
75	71	85.07	96	2.80	2.95	3.07	48	61.50	76	1.76	2.13	2.57
80	75	86.91	98	2.80	2.95	3.07	50	62.67	76	1.77	2.12	2.54
85	76	88.55	99	2.83	2.95	3.07	52	63.80	79	1.77	2.12	2.55
95	79	90.00	100	2.81	2.95	3.07	52	64.71	78	1.73	2.12	2.52
90	77	91.27	100	2.81	2.96	3.10	54	65.45	80	1.78	2.11	2.58
100	83	92.38	101	2.84	2.96	3.10	53	66.23	81	1.77	2.11	2.55

**Table 2:** Comparison between the default implementation and the greedy strategy of the 4${\textsc {SbIRT}}$ algorithm using the DS1 dataset

OP	Default implementation						Greedy strategy					
	Distance			Approx. ratio			Distance			Approx. ratio		
	Min.	Avg.	Max.	Min.	Avg.	Max.	Min.	Avg.	Max.	Min.	Avg.	Max.
05	05	10.17	15	1.25	2.37	3.00	4	5.61	13	1.00	1.33	2.75
10	11	20.54	29	1.57	2.64	3.00	8	12.68	23	1.00	1.63	3.00
15	19	29.81	38	1.91	2.74	3.10	13	19.94	30	1.15	1.83	2.80
20	28	38.11	49	2.14	2.80	3.08	16	26.52	37	1.33	1.95	2.69
25	33	45.37	58	2.35	2.83	3.07	23	32.16	43	1.39	2.01	2.71
30	39	51.91	66	2.53	2.86	3.00	27	37.10	49	1.55	2.04	2.58
35	45	57.58	70	2.48	2.88	3.00	30	41.29	53	1.58	2.06	2.58
40	49	62.69	77	2.62	2.90	3.00	34	44.90	56	1.62	2.07	2.53
45	54	67.19	81	2.70	2.91	3.00	37	48.07	60	1.69	2.08	2.55
50	58	71.11	83	2.71	2.92	3.04	39	50.68	63	1.69	2.07	2.50
55	61	74.56	87	2.72	2.92	3.04	41	53.07	64	1.65	2.08	2.50
60	64	77.64	92	2.73	2.93	3.00	43	55.11	68	1.74	2.08	2.46
65	69	80.32	93	2.79	2.93	3.00	44	56.85	71	1.72	2.07	2.44
70	71	82.68	96	2.79	2.94	3.04	46	58.43	72	1.73	2.07	2.46
75	72	84.84	95	2.79	2.94	3.04	47	59.83	72	1.76	2.07	2.48
80	75	86.70	98	2.80	2.94	3.00	49	61.01	73	1.77	2.07	2.46
85	76	88.30	99	2.81	2.94	3.00	51	62.11	76	1.74	2.07	2.43
95	78	89.75	99	2.83	2.95	3.03	51	63.06	75	1.73	2.07	2.45
90	78	91.00	100	2.83	2.95	3.00	52	63.80	77	1.73	2.06	2.45
100	82	92.13	99	2.83	2.95	3.03	54	64.54	76	1.75	2.06	2.43

**Table 3:** Comparison between the default implementation and the greedy strategy of the 3SbIRGT algorithm using the DS1 dataset

OP	Default implementation						Greedy strategy					
	Distance			Approx. ratio			Distance			Approx. ratio		
	Min.	Avg.	Max.	Min.	Avg.	Max.	Min.	Avg.	Max.	Min.	Avg.	Max.
05	5	9.89	14	1.25	2.30	2.80	4	5.46	12	1.00	1.29	2.75
10	11	20.19	28	1.57	2.59	2.89	7	12.34	22	1.00	1.59	2.83
15	19	29.41	38	1.91	2.70	2.92	13	19.53	30	1.15	1.80	2.70
20	27	37.68	48	2.14	2.77	2.94	16	26.06	36	1.27	1.91	2.67
25	33	44.94	57	2.29	2.80	2.95	22	31.66	42	1.39	1.98	2.64
30	40	51.47	66	2.50	2.83	2.95	27	36.57	48	1.55	2.01	2.53
35	44	57.13	70	2.48	2.85	2.96	30	40.75	53	1.58	2.04	2.53
40	49	62.24	77	2.64	2.87	2.96	34	44.33	56	1.64	2.04	2.53
45	54	66.73	80	2.68	2.88	2.96	37	47.54	60	1.67	2.05	2.50
50	59	70.66	82	2.67	2.89	2.96	39	50.12	63	1.68	2.05	2.48
55	61	74.10	87	2.71	2.90	2.97	41	52.52	65	1.62	2.05	2.50
60	63	77.17	90	2.76	2.91	2.97	43	54.56	67	1.70	2.05	2.46
65	68	79.85	92	2.79	2.91	2.97	44	56.31	70	1.70	2.05	2.44
70	71	82.21	95	2.78	2.91	2.97	46	57.84	71	1.69	2.05	2.46
75	71	84.38	94	2.79	2.92	2.97	47	59.26	72	1.72	2.05	2.43
80	74	86.21	97	2.80	2.92	2.97	49	60.43	72	1.70	2.05	2.43
85	75	87.83	98	2.77	2.92	2.97	51	61.54	75	1.73	2.05	2.42
95	78	89.28	98	2.81	2.93	2.97	51	62.48	74	1.71	2.05	2.41
90	77	90.54	100	2.83	2.93	2.97	52	63.21	76	1.73	2.04	2.42
100	82	91.65	99	2.80	2.93	2.97	52	63.95	76	1.72	2.04	2.40

**Table 4:** Results of the 4.5${\textsc {SbIRT}}$, 4${\textsc {SbIRT}}$, and 3SbIRGT algorithms considering the default implementation and the greedy strategy using the DS2 dataset

Size	Default Implementation						Greedy strategy					
	Distance			Approx. ratio			Distance			Approx. ratio		
	Min.	Avg.	Max.	Min.	Avg.	Max.	Min.	Avg.	Max.	Min.	Avg.	Max.
4.5SbIRT												
100	99	103.47	112	2.91	3.04	3.29	60	71.77	85	1.76	2.11	2.50
200	199	203.73	215	2.97	3.03	3.21	122	137.83	157	1.82	2.05	2.34
300	299	303.89	315	2.96	3.00	3.12	185	202.53	222	1.83	2.00	2.21
400	399	403.97	412	2.98	3.01	3.07	245	266.51	291	1.83	1.98	2.17
500	499	504.00	513	2.99	3.01	3.07	307	330.05	356	1.84	1.97	2.13
4SbIRT												
100	98	100.38	102	2.88	2.95	3.00	60	70.02	81	1.76	2.06	2.38
200	198	200.41	202	2.96	2.99	3.01	121	135.48	152	1.81	2.02	2.27
300	298	300.41	302	2.95	2.97	3.01	181	199.64	219	1.79	1.97	2.17
400	397	400.43	402	2.97	2.98	3.00	241	263.21	290	1.80	1.96	2.16
500	498	500.42	502	2.98	2.99	3.01	303	326.29	352	1.81	1.95	2.11
3SbIRT												
100	97	99.92	100	2.88	2.93	2.97	59	69.45	81	1.74	2.04	2.38
200	197	199.93	200	2.94	2.98	2.99	120	134.89	150	1.79	2.01	2.24
300	298	299.93	300	2.95	2.97	2.99	182	199.07	218	1.80	1.97	2.17
400	397	399.93	400	2.97	2.98	2.99	243	262.57	285	1.81	1.96	2.13
500	498	499.94	500	2.98	2.98	2.99	304	325.68	350	1.82	1.95	2.10

**Table 5:** The average running time of the 4.5${\textsc {SbIRT}}$, 4${\textsc {SbIRT}}$, and 3SbIRGT algorithms, in seconds, considering the default implementation (DI) and the greedy strategy (GS) using the DS2 dataset

Size	4.5${\textsc {SbIRT}}$		4${\textsc {SbIRT}}$		3SbIRGT	
	DI	GS	DI	GS	DI	GS
100	0.01	0.03	0.01	0.03	0.01	0.02
200	0.03	0.10	0.03	0.10	0.03	0.10
300	0.06	0.23	0.06	0.23	0.06	0.22
400	0.13	0.40	0.12	0.41	0.13	0.43
500	0.18	0.63	0.19	0.64	0.18	0.64

**Table 6:** Comparison between the $3{\textsc {SbI}}\overline{\text {R}}{\textsc {T}}{}$ and $4{\textsc {SbI}}\overline{\text {R}}{\textsc {T}}{}$ algorithms using the DB_SIRIT_ dataset

d	$3{\textsc {SbI}}\overline{\text {R}}{\textsc {T}}{}$		$4{\textsc {SbI}}\overline{\text {R}}{\textsc {T}}{}$		Small (%)	Small or equal (%)
	Distance (Avg.)	Approx. ratio (Avg.)	Distance (Avg.)	Approx. ratio (Avg.)		
10	12.60	1.68	12.19	1.63	51.37	68.70
20	26.37	1.77	25.60	1.71	54.72	66.48
30	40.23	1.81	36.91	1.66	78.60	85.53
40	53.29	1.85	46.10	1.60	95.84	97.62
50	63.78	1.87	53.21	1.56	99.46	99.74
60	71.88	1.89	58.97	1.55	99.81	99.92
70	77.83	1.90	63.29	1.55	99.97	99.98
80	82.45	1.91	66.73	1.55	99.95	99.99
90	85.98	1.92	69.48	1.55	99.97	99.99
100	88.78	1.93	71.64	1.56	99.99	99.99

**Table 7:** Comparison between the 2.5${\textsc {SbI}}\overline{\text {R}}{\textsc {GT}}$ and 3${\textsc {SbI}}\overline{\text {R}}{\textsc {GT}}$ algorithms using the DB_SIRGT_ dataset

d	2.5${\textsc {SbI}}\overline{\text {R}}{\textsc {GT}}$		3${\textsc {SbI}}\overline{\text {R}}{\textsc {GT}}$		Small (%)	Small or equal (%)
	Distance (avg.)	Approx. ratio (avg.)	Distance (avg.)	Approx. ratio (avg.)		
10	11.79	1.57	12.25	1.64	32.30	53.18
20	24.97	1.68	25.57	1.72	34.77	49.58
30	38.21	1.74	36.69	1.67	63.31	73.75
40	50.47	1.79	45.61	1.62	89.60	93.69
50	60.46	1.81	52.65	1.58	97.69	98.75
60	68.17	1.83	58.19	1.56	99.45	99.78
70	74.06	1.84	62.54	1.55	99.75	99.90
80	78.68	1.85	65.96	1.55	99.93	99.96
90	82.09	1.85	68.67	1.55	99.92	99.97
100	84.85	1.86	70.82	1.55	99.97	100.00

**Table 8:** Analysis of the topological characteristics of the phylogenetic trees generated by the results of the 2Sb$\overline{\text {R}}$ t and 3${\textsc {SbI}}\overline{\text {R}}{\textsc {GT}}$ algorithms compared with the phylogenetic tree presented by Laurence et al. [20]

	MAST	$I_{cong}$	P-value
2sb$\overline{\text {R}}$ t	46 Leaves	3.17	6.76e−22
3${\textsc {SbI}}\overline{\text {R}}{\textsc {GT}}$	51 Leaves	3.52	2.77e−25

From Fig. 6 (created using treeio R package [23]), we observe that the approach separates the organisms considering the species and performed good groupings. It is worth mentioning that the tree was based exclusively on rearrangement event information.

## Conclusion

We studied the sorting by intergenic reversals and transpositions problem on signed and unsigned permutations. We presented, for both cases, a 4-approximation algorithm, improving the 4.5 approximation factor previously known for the unsigned case. Besides, we generalized the transposition event and presented a 3-approximation algorithm to the problem that arises, which is more realistic in scenarios that consider intergenic regions. We developed a greedy strategy to improve the practical performance of the algorithms and conducted a comparison using datasets with different features. Considering the signed case of the problem, the tests indicated that our algorithms, in the vast majority of the cases, tend to provide better practical results compared with the previous known results. Moreover, we carried out an experiment using real genomes to verify the applicability of the proposed algorithms.

From the theoretical point of view, the algorithms proposed for the unsigned case of the sorting by intergenic reversals and transpositions problem bring an important improvement considering the approximation factor. On the other hand, the results for the signed case of the problem have the practical potential of enhancing the estimates for the distance of compared genomes, and consequently, the analysis regarding the genome rearrangements.

In future works, one can incorporate non-conservative events (e.g., insertion and deletion of genes or nucleotides) into the model.

## Data Availability

The algorithms and datasets generated during the current study are available in the following public repository: https://github.com/compbiogroup/Reversal-and-Transposition-Distance-Considering-Gene-Order-and-Intergenic-Sizes
